# Outcomes of Management of Severe Clubfoot among Children by Ilizarov External Fixator

**DOI:** 10.5704/MOJ.2311.003

**Published:** 2023-11

**Authors:** M Nwet, R Vignesh, W Khaing, S San, ZW Ko

**Affiliations:** 1 Surgical Based Department, Universiti Kuala Lumpur Royal College of Medicine Perak (UniKL RCMP), Ipoh, Malaysia; 2 Preclinical Department, Universiti Kuala Lumpur Royal College of Medicine Perak (UniKL RCMP), Ipoh, Malaysia; 3 Department of Pharmacotherapy, University of Utah, Salt Lake City, United States; 4 Department of Orthopaedics, University of Medicine, Yangon, Myanmar

**Keywords:** resistant clubfoot foot, Ilizarov frame, complex deformities

## Abstract

**Introduction:**

Clubfoot remains the most common birth defect involving the musculoskeletal system. There are various surgical and non-surgical treatment options available for the management of clubfoot. Using the minimally invasive Ilizarov external fixator method has been reported to have good success rates and fewer complications.

**Materials and methods:**

This study aimed at analysing the morphological and functional outcomes of treating severe clubfoot by Ilizarov external fixator among children from July 2017 to March 2020. Thirty-two children who had either failed Ponseti / surgery or neglected with 44 clubfeet of Dieglio type III and type IV were included in the study. A short-leg walking cast was applied for an additional six weeks after removing of Ilizarov frame and additionally followed by an orthosis for another six weeks. Outcomes were measured by the functional rating system by Laaveg and Ponseti and interpretation done at 1 month and 12 months after the ankle-foot arthrosis.

**Results:**

About 86.4% of the patients had good or excellent outcome scores. Pre and post-Demeglio scores and functional rating scores were statistically significant (p<0.001) by using Paired t-test. Complications included superficial pin site infections in 13 feet (29.54%), 5 feet (11.36%) had claw toes, 3 feet (6.81%) had linear skin necrosis and 2 feet (4.54%) had calcaneal fractures which were manageable with minor interventions.

**Conclusion:**

The study findings highlighted that the Ilizarov external fixator method can correct complex foot deformities of severe clubfoot with minimum morbidity. Further larger and long-term studies are needed to investigate the effects of the stiff hindfoot and possible degenerative changes on the function and symptoms of these patients as adults.

## Introduction

Congenital Talipes Equinovarus (CTEV) or idiopathic clubfoot is one of the most common congenital abnormalities involving the musculoskeletal system with an incidence of about 1-6 in every 1000 live births^1^. Classical CTEV deformities include ankle equinus, hindfoot varus, midfoot supination, forefoot adductus, and variable degree of cavus. Although clubfoot can be easily diagnosed by a general practitioner or trained midwife, accurate classification and assessment of underlying pathology would require the expertise of an experienced clinician^2^. There are two common methods of evaluation for CTEV, namely the Pirani and Dimeglio classifications^3,4^. Dimeglio classification relies on the passive range of motion in four planes of the foot, where grades III and IV are the most severe forms and are associated with the least satisfactory outcome. In many developing countries, children with severe CTEV presenting late after walking age are still common despite the advancement of information technology, and their management remained a challenge to the orthopaedic community^4^.

Many methods of treatment for CTEV have been reported over the years. For the non-surgical treatment, Kite and Mackay popularised serial casting during the 70s and 80s of the last centuries^5-7^. The French daily strapping method was introduced in 19905. Ponseti and his team reported good treatment outcomes using his serial casting protocol for CTEV children under two years of age^8^. However, for children presenting later than two years, the correction might be difficult because of contracted skin, tendons, ligaments and capsules on the posterior medial aspect of the clubfoot^9,10^.

For severe CTEV that failed non-operative treatment, there are also many surgical options, intending to provide plantigrade and pain-free feet. One of the first operations was described by Phelps in 1891. Turco popularised the extensive posteromedial release in 198011. Because of the patho-anatomy of CTEV, acute correction of all the deformities would require extensive dissection of the foot that could result in a stiff foot due to fibrosis and scarring. In addition, there is a significant risk of neurovascular injury, wound dehiscence residual deformity, muscle weakness and shortened foot^12^.

In the 1950s Professor Gavil Abramovich Ilizarov from Russia developed the Ilizarov apparatus to perform distraction histogenesis. The ring external fixation is also used to treat non-union, and correct soft tissue deformities, including clubfoot^12^.

There are a few different techniques for treating clubfoot deformity with an Ilizarov external fixator. It varies from acute soft tissue correction and fixation with the Ilizarov device, a combination of osteotomy / soft tissue release and gradual stretching, and complete gradual stretching of soft tissue without any acute release or lengthening. Ilizarov external fixator has the potential to perform differential stretching of soft tissue in different regions of the foot, allowing three-dimensional correction of the complex foot deformities^13-16^. Most publications on the treatment of CTEV using Ilizarov external fixator were small case series with no clear description of the actual frame configuration.

We conduct this study to evaluate the treatment outcome of resistant CTEV using a pre-determined Ilizarov external fixator configuration that corrected all components of foot deformities without osteotomy or soft tissue surgery.

## Materials and Methods

This is a prospective clinical study conducted at Children Hospital from January 2017 to March 2020. Ethical approval was obtained from the ethical board of the University of Medicine and Children Hospital. Written consent was obtained from the parents of all the children enrolled in this study. Only children aged between two and twelve were recruited for the study.

**Fig 1:**
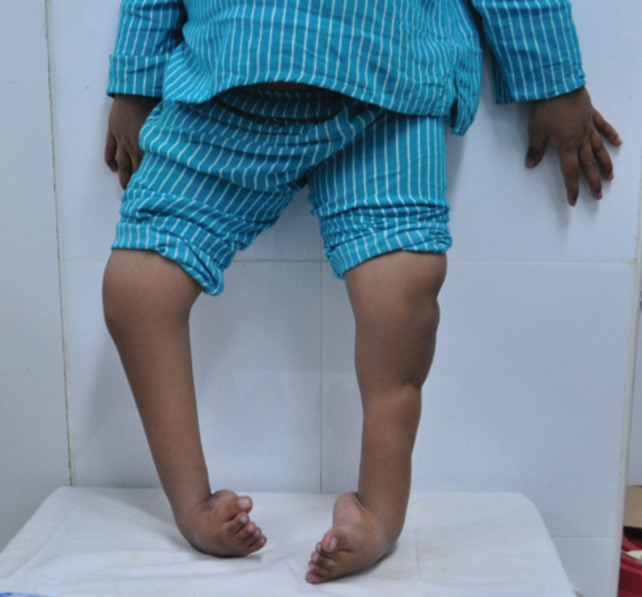
Five-year-old child with bilateral clubfoot.

We recruited all children diagnosed with CTEV or idiopathic clubfoot (unilateral and bilateral) who had failed Ponseti treatment/surgery and were neglected. Children who presented with non-healing ulcers over the lateral foot column and those with underlying neuromuscular abnormalities were excluded from the study. After admission to the ward, we collected the demographic data and assessed the condition based on Functional rating scores and Dimeglio scores^2,17,18^. Fig. 1 shows a five-year-old child with bilateral clubfoot classified as Dimeglio type IV. We did not routinely perform pre-operative radiographs for the foot unless we suspected underlying congenital bone abnormality.

The procedures were performed by two senior orthopaedic surgeons for all the cases. Based on the size of the foot, appropriate size half rings were used to construct a fully constrained Ilizarov frame following a standard configuration [SH Pitkar, Orthotools, Pune, India]. The Ilizarov frame was pre-assembled and adjusted to match the degree of foot deformity in all the plains. As the first step, the calcaneus was fixed to a one-half ring using two plain stainless-steel wires of 1.8mm, with special attention to placing the hinge along the bimalleolar axis of the ankle. This was followed by fixing the first and fifth metatarsus each with one plain stainless-steel wire to another half-ring over the forefoot. Finally, two transverse wires and one or two 3.5/4.5mm half pins were used to fix the rings over the distal tibia bone. Additional components and fixation elements can be added whenever necessary. The front end of the calcaneal half-ring was fixed to biplanar hinges that extended proximally to the tibia ring(s) with one medial and another lateral threaded rod. At the same end of the calcaneus half-ring, two threaded rods extended anteriorly to connect the metatarsus half-ring over the forefoot^19^.

**Fig 2:**
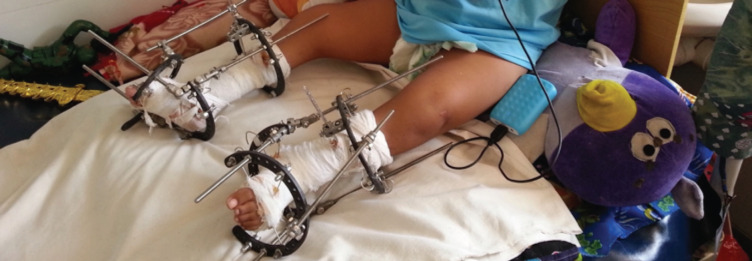
Five-year-old child with Ilizarov external fixator.

Sagittal plane correction (equinus) was achieved through an additional shortening element connecting the tibia ring to the forefoot half ring. Coronal plane correction (varus/valgus) of the hindfoot was corrected by distracting elements over the medial threaded rods connecting the calcaneus half ring and tibia ring. Both the corrections occurred through the biplanar hinges. Correction of forefoot adduction and cavus deformity was achieved by a distracting element over the medial threaded rod connecting the calcaneus half ring and forefoot half ring^19^. Fig. 2 shows the five-year-old child with Ilizarov external fixator. There was no additional procedure in the form of bone or soft tissue surgery other than what has been described.

The correction started with the gradual distraction of the medial threaded rods across the foot. At the same time, hindfoot varus was corrected by the distraction of the medial threaded rods across the ankle, while ankle equinus deformity corrected by shortening the additional element located over the dorsal aspect of the foot. The corrections can be started on the second post-operative day, at the rate of about 2-3mm a day. The rate of equinus correction can sometimes be increased to 4-5mm shortening a day. During the distraction, tension over the soft tissues, neurovascular status and improvement in the correction of the deformities were observed. Adjustments were performed by the surgeon and post-graduate students during their stay in the hospital.

All the pin wounds were cleaned with normal saline and dressed with sterilised dry gauze initially after 24 hours and then after 72 hours. Loose clamps, nuts and bolts were tightened regularly. The deformity was corrected weekly. The actual rate for deformity correction may vary depending on the resistance and rigidity of the deformities.

We always looked for complications and tried to resolve them as soon as possible. After complete correction, the Ilizarov frame was retained in the final position for a period of three weeks and patients could go back home after their parents or caretakers were trained to take care of pins and regularly tightened any loose clamps, nuts and bolts. During this period, the patients were encouraged and allowed to bear full weight and walk normally. After the Ilizarov fixator was removed, a short leg plaster of Paris cast was applied for two weeks. The cast was removed in the outpatient clinic and the status of the wounds was checked. New plaster of Paris cast was applied for another four weeks. The total time of plaster of Paris short cast was six weeks after removal of the Ilizarov external fixator, followed by an ankle-foot orthosis (AFO) for six weeks. All the patients were assessed regularly every month after finishing foot orthosis.

Pre-operative and outcome were assessed with Dimeglio score^17^. Sagittal plane evaluation of equinus, frontal plane evaluation of varus, horizontal plane evaluation of derotation of the calcaneopedal block and horizontal plane evaluation of forefoot relative to hindfoot was assessed. The outcome measure used to assess foot function was the Functional Rating System for clubfoot (FRS) of Laaveg and Ponseti^16^. Excellent score is 90 to 100, Good score is 80 to 89, the Fair result is 70 to 79 and Poor, is less than 70. The outcome measurements were performed, as the mean of one month and twelve months after the completion of the foot orthosis using the Functional Scoring System (Table I).

**Table I:** Functional Rating System (FRS)^16^

Category	Points
**Satisfaction** (20 points)	
I am …..	
1 Very satisfied with the end result	20
2 Satisfied with the end result	16
3 Neither satisfied nor unsatisfied with the end result	12
4 Unsatisfied with the end result	8
5 Very unsatisfied with the end result	4
Function (20 points)	
In my daily living, my club foot….	
1 Does not limit my activities	20
2 Occasionally limits my strenuous activities	16
3 Usually limits me in strenuous activities	12
4 Limits me occasionally in routine activities	8
5 Limits me in walking	4
Pain (30 points)	
My club foot….	
1 Is never painful	30
2 Occasionally causes mild pain during strenuous activities	24
3 Usually is painful after strenuous activities only	18
4 Is occasionally painful during routine activities	12
5 Is painful during walking	6
Position of heel when standing (10 points)	
1 Heel varus, 0 or some heel valgus	10
2 Heel varus, 1 - 5 ֩	5
3 Heel varus, 6 - 10 ֩	3
4 Heel varus, greater than 10 ֩ Passive motion (10 points)	0
1 Dorsiflexion	1 point per 5
(up to 5 points)	
2 Total varus-valgus motion of heel	1 point per 10
(up to 3 points)	
3 Total anterior inversion-eversion of foot	1 point per 25
(up to 2 points)	
Gait (10 points)	
1 Normal	6
2 Can toe-walk	2
3 Can heel- walk	2
4 Limp	-2
5 No heel strike	-2
6 Abnormal toe-off	-2

Notes: Poor: <70, Fair: 70 – 79, Good: 80 – 89, Excellent: 90 – 100

Data were analysed with the Statistical Package for Social Sciences software version 25.0 [IBM Corporation, Armonk, NY, USA]. First, descriptive statistics were done. Then, the paired sample t-test was used to determine pre- and post-intervention Dimeglio score and FRS. P-value <0.05 was considered statistically significant.

## Results

We recruited 32 children with 44 clubfeet who fulfilled the inclusion criteria. The mean duration for the application of Ilizarov external fixator was 11.84±1.52 weeks, ranging from a minimum of 9 weeks to a maximum of 15 weeks.

The pre-operative mean Dimeglio score was 15.82±1.64, and it improved to 4.27±0.45 after the operation. Based on paired sample t-test analysis, the difference was statistically significant (p<0.001).

According to the Laaveg and Ponseti functional rating system, before treatment with Ilizarov external fixator, under the parent or patient-reported outcome at the one month of follow-up after removing of ankle-foot orthosis was applied, the mean satisfaction score (16.00 ±0.00) and the mean functional score (17.3±1.88) were both low (maximum of 20 points). The mean pain score (24.7±1.93) was also low (maximum of 30 points) suggesting that most of the feet were painful during routine walking. Under the pre-operative physical evaluation, all the clubfeet had varus heel of greater than 10° with the mean score of 5.9±2.27 (maximum of 10 points). The mean score for the passive motion was 7.5±1.85 (maximum of 10 points), and the mean score for gait was 6.3±2.20 (maximum of 10 points), suggesting a limping gait with abnormal toe-off and heel strike.

Clinical review of 12 months, most of the patients and their parents were very satisfied with the results. The mean satisfaction score was 20, and the mean functional score was 18.88±1.85. For pain, the mean score was 28.2±2.77, indicating that most of the club feet were occasionally painful during strenuous activities. Under the physical evaluation, most of the heel position was in 0o varus, with the mean heel position score of 5.9±2.27. The mean passive motion score improved to 7.5±1.85, while the mean gait score also improved to 6.3±2.20. (Table II).

**Table II:** Comparison of the mean of pre-operative and 1 month and 12 month post-operative functional rating system score

FRS Score (Mean+SD)	Pre-operative	Post-operative (1 month)	Post-operative (12 month)
Satisfaction	5.0±1.75	16.0±.00	20.0±0.00
Function	9.5±1.95	17.3±1.88	18.8±1.85
Pain	9.7±3.60	24.7±1.93	28.2±2.77
Heel Position	0.0±000	5.9±2.27	5.9±2.27
Passive Motion	3.2±1.03	7.5±1.85	7.5±1.85
Gait	-6.0±.000	6.3±2.20	6.3±2.20

The mean duration of follow-up was 16.8±4.03, ranging from the shortest duration of 12 months to the longest duration of 22 months. The outcome assessments were performed one month and 12 months after treatment. The outcome of 12 feet was rated as excellent, 26 feet as good and 6 as fair. There was no poor outcome after treatment.

The correlation between one-month follow-up and 12 months follow-up of satisfaction, function and pain also showed improved results. Statistical analysis was not performed since the heel position, passive motion and gait score are not changed. Therefore, based on the observations, we interpret that there is no relapse. For the age group and FRS of 12-month follow-up post-cross-tabulation, there was no significant association (Chi-square p-value=0.164, Fisher’s Exact p-value=0.163) observed.

A chi-square test of independence was conducted to determine the link between the age group (<=5, 6-10, >10) and the final FRS score group (excellent, good, fair). There was no association between the two variables that were statistically significant. (χ^2^(4) = 6.188, p-value = 0.164, Fisher's exact p-value = 0.163). Additionally, a chi-square test of independence was conducted between the previous condition (neglected, relapsed) and the final FRS outcome group (excellent, good, fair). No association was statistically significant between the two variables. (χ^2^(2) = 4.642, p-value = 0.103, Fisher's exact p-value = 0.126) In addition, a chi-square test of independence was conducted between the preoperative Dimeglio score (Dimeglio III and Dimeglio IV) and the final FRS outcome group (excellent, good, fair). No association was statistically significant between the two variables. (χ^2^(2) = 0.342, p-value = 0.913, Fisher's exact value = 0.913).

In this study, superficial pin tract infection developed in 13 clubfeet (29.54%). There was no incidence of deep pin tract infection like abscess or osteomyelitis. We managed these infected wounds by applying dressings with local antiseptic solution (Chlorhexidine / Betadine) daily and prescribed oral broad-spectrum antibiotics. Clawing of toes was noted in 5 feet (11.36%) during the distraction period due to stretching of the flexor tendons of the toes. They were more common in older children. We could temporarily stop the distraction, and either perform frequent stretching of the affected toes or fix the toes to the metatarsus in an extended position. The gradual correction would resume in about one week. In 3 feet (6.81%) we noted linear skin necrosis over the medial aspect of the feet between the pin sites of the first metatarsus and medial calcaneus. We temporarily stopped the distraction, debrided the wound and prescribed antibiotics. There were two cases of calcaneal fracture due to the Kirschner wire cutting through the bone. After radiology confirmation, distraction was temporarily slowed down until an additional Kirschner wire was inserted to secure the calcaneus.

## Discussion

Our aim for treating CTEV is to achieve a pliable, functional and painless foot. Although there had been many reports of good results following treatment with extensive surgical release, the treated feet were generally shorter with various degrees of stiffness or ankylosis, predisposing to degenerative arthritis, especially of the mid and hindfoot^20^. The long-term outcome evaluation showed very limited ankle movements, and this was closely related to the degree of talar dome flattening^21^.

In this study, we treated a group of walking-age children with severe clubfeet (Dimeglio III and IV) by gradual soft tissue distraction using the Ilizarov external fixator without the need to perform bone osteotomy or soft tissue procedures like surgical release or tendon lengthening. The Ilizarov technique has been used to treat many types of complex foot deformities in children, properly constructed Ilizarov external fixator allows simultaneous correction of deformities in all three orthogonal planes^22^. The frame is strong enough to distract most of the stiff and contracted soft tissues. With gradual distraction, it can reduce the risk of injury to the blood vessels, nerves, muscles, tendons, and skin. There is also the possibility of lengthening a short foot^23^.

Bradish *et al* reported the Ilizarov method was used to treat 17 relapsed club feet in 12 children with a mean age of 7.8 years. They achieved excellent and good results in 13ft based on evaluation at a 3-year follow-up^24^. In our study the mean age was the same as clinical research done by Bradish. In our country, the school-going age started from five years of age and when they went to school, their friends and the environment started to notice this obvious deformity of clubfoot and tease them daily. That had been the main reason for them and their caregivers to seek medical advice for correction of those feet.

The study group of Barbary included 66 feet in 52 patients (mean age 8.5years) who were graded as severe Dimeglio classification grade III and 7ft grade IV. The results were good with a mean follow-up of 40 (26-58) months^25^. Utukuri grade D (Dimeglio-Bensahel system) 26 feet in 23 children of average 9 years followed-up 47 had a good result (52% Excellent and good) 57% Cosmetic^26^. In our study, most of the patients had grade IV deformities.

Salama *et al* had reported a 70% good results in 3 to 7 years clubfoot patients who had applied the fixation for 6 to 8 weeks^27^. In our study, all patients had a plantigrade foot by applying the Ilizarov frame for an average of 11 weeks and this was the same as that of Ferreira research^28^.

The aim of our treatment is to gain a fully plantigrade, painless mobile foot using the bloodless technique and it was in same as the study by Hosny and Makhdoom^29,30^. Wallander reviewed the good outcome in 10 clubfeet in 7 patients with a median of 40 (25-56) months follow-up. Six patients were satisfied, and most of the patients got better walking^31^. Leonchuk reported that about 93.5% had the desired results for 7 to 17 years of 108 patients with 126ft treated with Ilizarov external fixator^32^. El-Adly and Mostafa reported that an average of 9 years of 15 patients had to achieve the required correction within an average of 36 months of follow-up^33^. Gopinathan *et al* reported that an eight-year-old child with a residual club foot attained good result after being treated using the Ilizarov frame application^34^. In our study, all patients had good results, and in agreement with other similar studies.

Leonchuk studied 108 patients (126ft) aged between 7 and 17 years (mean age was 12.2±2.3) with Ilizarov external frame treatment and 22 cases got complications. All complications were transosseous osteosynthesis and external frame. All complications did not influence the final result. All pin tract infections can be cured with local care and oral antibiotics however pin-tract infection could not be ignored because of getting sepsis and losing the fixation^32^. In our study, the complications were manageable and did not interfere with overall patient satisfaction in the treatment of resistant clubfeet with an Ilizarov external fixator. In the course of the treatment, the complications were superficial pin tract infection, claw toes, lineal skin necrosis and calcaneal fracture at the wire site. The most common immediate complications observed in the current series were superficial pin tract infection and clawing of the toes.

All the patients of clubfeet were provided with the hospital stay, food and treatment free of charge. The inpatient treatment allowed close monitoring of the soft-tissue status and prevention of severe complications. A long hospital stay may avoid additional costs for additional procedures^23^.

The long-term effects have not been studied in the present article because all patients could not be followed up until maturity. As the growth potential is still present in these patients, it may be meaningful to observe the progression until maturity. The follow-up period was short in this study and so long-term results need to be assessed such as pain, stiffness, disabling deformity and functional gait.

## Conclusion

This study showed that the Ilizarov external fixator technique corrected deformities and achieved good functional and morphological results with no recurrences within 12 months. The findings also show that this method can correct severe clubfoot deformities with minimal morbidity, although larger and longer-term studies are needed to investigate the impact of the stiff hind foot and possible degenerative changes on the function and symptoms of these patients in adulthood.

## References

[1] Anand A, Sala DA (2008). Clubfoot: Etiology and treatment.. Indian journal of orthopaedics..

[2] Nguyen MC, Nhi HM, Nam VQD, Thanh DV, Romitti P, Morcuende JA (2012). Descriptive epidemiology of clubfoot in Vietnam: a clinic-based study.. Iowa Orthop J..

[3] Dimeglio A, Bensahel H, Souchet P, Mazeau P, Bonnet F (1995). Classification of clubfoot.. J Pediatr Orthop B..

[4] Bor N, Herzenberg JE, Frick SL (2006). Ponseti management of clubfoot in older infants.. Clin Orthop Relat Res..

[5] Islam ANMM, Ahmad SA, Kabir MR, Siddique MK, Arefin A (2016). Effectiveness of French Physiotherapy in Treating Congenital Clubfoot Deformity.. Ortho & Rheum Open Access J..

[6] Beaty JH, Canale ST, Beaty JH (2007). Congenital anomalies of the lower extremity.. Campbell’s Operative Orthopaedics..

[7] Sud A, Tiwari A, Sharma D, Kapoor S (2008). Ponseti's vs. Kite's method in the treatment of clubfoot--a prospective randomised study.. Int Orthop..

[8] Matuszewski L, Gil L, Karski J (2012). Early results of treatment for congenital clubfoot using the Ponseti method.. Eur J Orthop Surg Traumatol..

[9] Dobbs MB, Gurnett CA (2009). Update on clubfoot: etiology and treatment.. Clin Orthop Relat Res..

[10] Ponseti IV, Zhivkov M, Davis N, Sinclair M, Dobbs MB, Morcuende JA (2006). Treatment of the complex idiopathic clubfoot.. Clin Orthop Relat Res..

[11] Singh BI, Vaishnavi AJ (2005). Modified Turco procedure for treatment of idiopathic clubfoot.. Clin Orthop Relat Res..

[12] Spiegelberg B, Parratt T, Dheerendra SK, Khan WS, Jennings R, Marsh DR (2010). Ilizarov principles of deformity correction.. Ann R Coll Surg Engl..

[13] Fernandes RM, Mendes MD, Amorim R, Preti MA, Sternick MB, Gaiarsa GP (2016). Surgical treatment of neglected clubfoot using external fixator.. Rev Bras Ortop..

[14] Freedman JA, Watts H, Otsuka NY (2006). The Ilizarov method for the treatment of resistant clubfoot: is it an effective solution?. J Pediatr Orthop..

[15] Paley D (1993). The correction of complex foot deformities using Ilizarov's distraction osteotomies.. Clin Orthop Relat Res..

[16] Lykissas MG, Crawford AH, Eismann EA, Tamai J (2013). Ponseti method compared with soft-tissue release for the management of clubfoot: A meta-analysis study.. World J Orthop..

[17] Andriesse H, Roos EM, Hagglund G, Jarnlo GB (2006). Validity and responsiveness of the Clubfoot Assessment Protocol (CAP). A methodological study.. BMC Musculoskelet Disord..

[18] Sætersdal C, Fevang JM, Bjørlykke JA, Engesæter LB. (2016). Ponseti method compared to previous treatment of clubfoot in Norway. A multicenter study of 205 children followed for 8-11 years.. J Child Orthop..

[19] Kirienko A, Villa A, Calhoun JH (2003). Ilizarov Technique for Complex Foot and Ankle Deformities..

[20] Bocahut N, Simon AL, Mazda K, Ilharreborde B, Souchet P (2016). Medial to posterior release procedure after failure of functional treatment in clubfoot: a prospective study.. J Child Orthop..

[21] Hutchins PM, Foster BK, Paterson DC, Cole EA (1985). Long-term results of early surgical release in club feet.. J Bone Joint Surg Br..

[22] Malizos KN, Gougoulias NE, Dailiana ZH, Rigopoulos N, Moraitis T (2008). Relapsed clubfoot correction with soft-tissue release and selective application of Ilizarov technique.. Strategies Trauma Limb Reconstr..

[23] Ferreira RC, Costo MT, Frizzo GG, da Fonseca Filho FF (2006). Correction of neglected clubfoot using the Ilizarov external fixator.. Foot Ankle Int..

[24] Bradish CF, Noor S (2000). The Ilizarov method in the management of relapsed club feet.. J Bone Joint Surg Br..

[25] El Barbary H, Abdel Ghani H, Hegazy M (2004). Correction of relapsed or neglected clubfoot using a simple Ilizarov frame.. Int Orthop..

[26] Utukuri MM, Ramachandran M, Hartley J, Hill RA (2006). Patient-based outcomes after Ilizarov surgery in resistant clubfeet.. J Pediatr Orthop B..

[27] Salama KS, El-Adawy MF, Samaan SS, Rakha II (2017). Evaluation of ilizarov role in correction of relapsed clubfoot.. Egypt Orthop J..

[28] Ferreira RC, Costa MT, Frizzo GG, Santin RA (2007). Correction of severe recurrent clubfoot using a simplified setting of the Ilizarov device.. Foot Ankle Int..

[29] Hosny GA (2002). Correction of foot deformities by the Ilizarov method without corrective osteotomies or soft tissue release.. J Pediatr Orthop B..

[30] Makhdoom A, Qureshi PA, Jokhio MF, Siddiqui KA (2012). Resistant clubfoot deformities managed by Ilizarov distraction histogenesis.. Indian J Orthop..

[31] Wallander H, Hansson G, Tjernstrom B (1996). Correction of persistent clubfoot deformities with the Ilizarov external fixator. Experience in 10 previously operated feet followed for 2-5 years.. Acta Orthop Scand..

[32] Leonchuk SS, Neretin AS, Shestsov VI (2017). Complications in treatment of older children with congenital clubfoot by Ilizarov external fixator.. J Orthop Trauma Surg Rel Res..

[33] El-Adly WY, Mostafa KM (2009). Ilizarov external fixator in treatment of severe recurrent congenital talipes equinovarus.. Eur J Orthop Surgery Traumatol..

[34] Gopinathan NR, Rangasamy K, Sharma S, Sudesh P (2020). Ilizarov Frame Application Based on Ponseti Principles for Clubfoot Correction: A Case Report and Description of Surgical Technique.. Indian J Orthop..

